# Acetate uptake alleviates propionate-mediated growth restriction in *Yersinia enterocolitica*

**DOI:** 10.1128/iai.00043-26

**Published:** 2026-04-15

**Authors:** Matthew K. Muramatsu, Dylan Cole, Sebastian J. Perez-Orozco, Sebastian E. Winter

**Affiliations:** 1 Department of Internal Medicine, Division of Infectious Diseases, UC Davis School of Medicine, Davis, California, USA; 2 Department of Medical Microbiology and Immunology, UC Davis School of Medicine, Davis, California, USA; University of California San Diego School of Medicine, La Jolla, California, USA

**Keywords:** host-microbe interaction, SCFA toxicity, *Yersinia enterocolitica*

## Abstract

The gut microbiota impedes infection by enteric pathogens, a process termed colonization resistance. Microbial production of short-chain fatty acids (SCFAs), such as acetate, propionate, and butyrate, contributes to colonization resistance. *Yersinia enterocolitica* encounters short-chain fatty acids at several stages during intestinal infection. However, our understanding of how *Y. enterocolitica* copes with SCFA stress is limited. Here, we found that acetate, propionate, and butyrate restrict *Y. enterocolitica* growth *in vitro*. Propionate exerted the most potent toxicity by both pH-dependent and pH-independent mechanisms. pH-dependent propionate growth restriction was worsened in a mutant lacking ornithine decarboxylase, suggesting that this enzyme is involved in counteracting cytoplasmic acidification by propionate under acidic environmental conditions. pH-independent propionate toxicity required phosphate acetyltransferase (phosphotransacetylase) and acetate kinase, pointing to conversion of intracellular propionate to toxic propionyl-CoA by promiscuous phosphotransacetylase and acetate kinase activities as a mechanism of propionate toxicity. We also found that pH-independent propionate toxicity was alleviated by exogenous acetate, taken up via the acetate/succinate transporter SatP. This work advances our understanding of how short-chain fatty acids restrict pathogen growth and highlights strategies used by bona fide pathogens to overcome short-chain fatty acid-mediated colonization resistance.

## INTRODUCTION

Human infection with *Yersinia enterocolitica* results in a spectrum of clinical manifestations, ranging from self-limiting gastroenteritis (1, 2) to severe systemic infection. Immunocompromised patients (3) or patients with hemochromatosis (4, 5) are at increased risk of systemic dissemination of *Y. enterocolitica*. In rare cases, transfusion-associated bacteremia can occur (6). Humans contract *Y. enterocolitica* by the fecal-oral route, primarily through the consumption of contaminated food products from animal reservoirs (7–9). This is particularly concerning from a food safety perspective because, unlike other enteric pathogens, it exhibits a notable cold tolerance (10, 11).

Upon ingestion, *Y. enterocolitica* traverses the intestinal tract until it reaches the ileum and colon (12–14), where it encounters a dynamic microenvironment populated by diverse microbial communities. These microbial communities work with the host to inhibit the colonization of potentially harmful microorganisms, a phenomenon termed colonization resistance (15, 16). Microbiota-derived short-chain fatty acids (SCFAs) are a key component of colonization resistance (17, 18). SCFAs are generated from anaerobic fermentation, with the most abundant forms being acetate, butyrate, and propionate (19–21). Disruption of gut microbial communities, e.g., by oral antibiotics, results in the diminished production of SCFAs and sensitivity to colonization by professional and opportunistic pathogens, such as multi-drug resistant/carbapenem-resistant Enterobacteriaceae (22, 23).

Commensal microorganisms in the mammalian gut produce butyrate and propionate from structurally diverse complex polysaccharides (24). Members of the *Bacteroidetes* phylum metabolize hexose and pentose sugars through the succinate pathway to produce SCFAs, while members of the *Lachnospiraceae* can metabolize deoxy sugars (fucose and rhamnose) through the propanediol pathway to generate propionate (25). *Lactobacillus*, commonly found in the small and large intestines, can also use exogenous 1,2-propanediol to produce propionate and propanol, indicating that indirect cross-feeding could also be an important route of propionate generation (26). Butyrate production is performed primarily by members of the *Bacillota* (*Firmicutes*) phylum. Four distinct metabolic pathways—the acetyl-CoA pathway, the glutarate pathway, the 4-aminobutyrate pathway, and the lysine pathway—contribute to overall butyrate production (27). In the human gut, total SCFA concentrations can range from 50 to 200 mM. Based on stool sampling, the average concentration of propionate is around 21 mM (28–31).

SCFAs inhibit the growth of many microorganisms (23, 32–35). In principle, two general mechanisms of direct SCFA toxicity have been proposed to explain the SCFA-mediated colonization resistance against enteric pathogens. The first mechanism is based on the accumulation of toxic metabolite(s) as part of propionate degradation (36). For example, *Salmonella enterica* serovar Typhimurium degrades propionate via the methylcitrate cycle (MCC) (37). In the MCC, oxaloacetate and propionate (activated as propionyl-CoA) are converted to pyruvate and succinate (38). Accumulation of 2-methyl citrate, an intermediate of the MCC, inhibits the gluconeogenic enzyme, fructose 1,6 bisphosphatase, thus limiting growth (39). Curiously, the MCC pathway in *Y. enterocolitica* is predicted to be incomplete. The second mechanism involves acidification of the cytoplasm. Under acidic environmental conditions, the equilibrium of protonated and deprotonated SCFAs is shifted towards the protonated form, which can traverse cell membranes (40). Upon reaching the neutral cytoplasm, SCFAs deprotonate, and the uncontrolled influx acidifies the cytoplasm of Enterobacteriaceae (22), inhibiting their growth. *Y. enterocolitica* encounters SCFAs as it colonizes the intestinal tract. However, it is unclear whether SCFAs inhibit growth of *Y. enterocolitica* and how *Y. enterocolitica* overcomes any potential toxic effects exerted by SCFAs.

Here, we report that propionate inhibits growth of *Y. enterocolitica in vitro*. The mechanism of toxicity likely involves promiscuous activities of acetate kinase (AckA) and phosphate acetyltransferase (Pta). AckA and Pta convert propionate to propionyl-CoA, which in turn may inhibit key physiological functions. We also found that exogenous acetate, taken up by the transporter SatP, prevents accidental conversion of propionate to propionyl-CoA, thus rescuing propionate toxicity.

## RESULTS

### Acetate, propionate, and butyrate restrict growth of *Y. enterocolitica in vitro*

**Fig 1:**
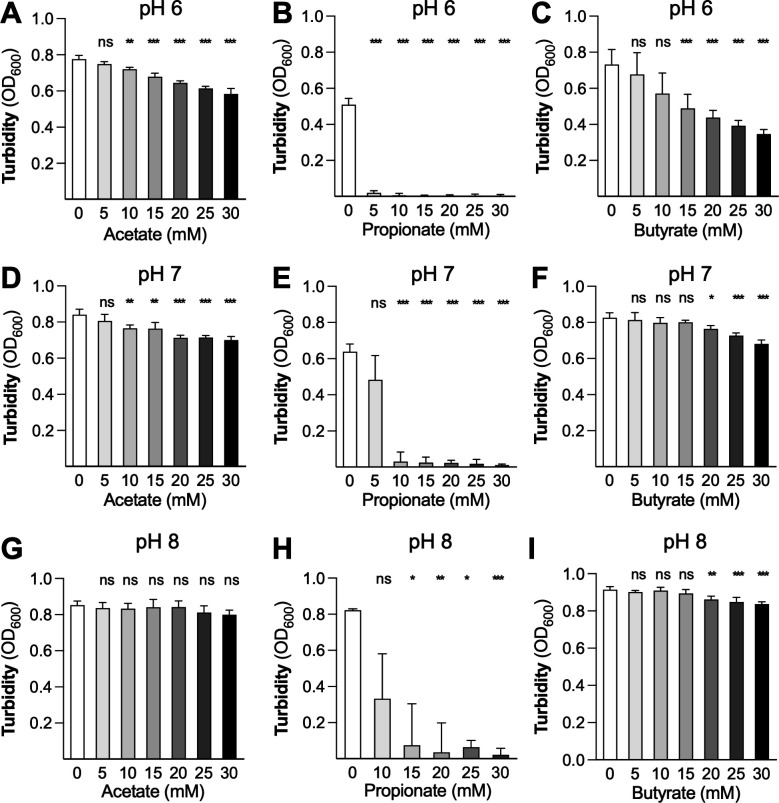
Effect of acetate, propionate, and butyrate on *Y. enterocolitica* growth *in vitro*. The *Y. enterocolitica* wild-type strain WA-314 was cultured in LB in the presence of increasing concentrations of sodium acetate (**A, D, and G**), propionate (**B, E, and H**), or butyrate (**C, F, and I**), at a starting pH of 6 (**A–C**), pH of 7 (**D–F**), or pH of 8 (**G–I**). Cultures were incubated for 24 h at 37°C. The absorbance of the culture at 600 nm (OD_600_) was then determined. Bars represent the geometric mean, and error bars represent the geometric standard deviation from four independent experiments. Statistical significance is indicated above each bar, using untreated cells as a comparison. ns, not statistically significant; *, *P* < 0.05; **, *P* < 0.01; ***, *P* < 0.001. Log-normal, one-way ANOVA/Dunnett’s multiple comparison test.

To begin to understand how SCFAs impact *Y. enterocolitica* replication, we cultured *Y. enterocolitica* WA-314 (serotype O:8) in the presence of acetate, propionate, and butyrate and quantified growth (Fig. 1). Since the concentration of SCFAs varies in the gut, we used a dose-response assay using a physiological range of concentrations from 5 to 30 mM (41, 42). Since extracellular pH is known to influence the toxicity of SCFAs, we adjusted the pH of the starting media to pH values of 6, 7, and 8, reflecting the pH range of different regions in the human terminal ileum (pH 7.0–7.5), cecum (approximately pH 6), and colon (pH increasing from 6.5 in the ascending colon to up to pH 8.0 in the sigmoid colon) (43–45). We incubated the cultures at 37°C, approximating human body temperature, and measured the turbidity of the culture (OD_600_) after 24 h as a proxy for growth. At pH 6, acetate, propionate, and butyrate restricted *Y. enterocolitica* growth in a dose-dependent fashion (Fig. 1A through C). Propionate showed the most prominent effect, essentially preventing growth at a concentration of 5 mM and higher (Fig. 1B).

When the pH outside the cell is lower than pH 7.5–7.7, the normal cytoplasmic pH of neutrophilic bacteria (46–48), increased diffusion of SCFA into the bacterial cell leads to cytoplasmic acidification (22). Consistent with this idea, the magnitude of growth inhibition by acetate, propionate, and butyrate decreased when the pH was increased to 7 (Fig. 1D through F) and 8 (Fig. 1G through I). Acetate lost virtually all activity at pH 8 (Fig. 1G), while butyrate still exhibited a moderate degree of toxicity (Fig. 1I). In contrast, propionate markedly inhibited *Y. enterocolitica* growth even at pH 8 (Fig. 1H). These results suggest that acetate and butyrate impede *Y. enterocolitica* growth primarily in a pH-dependent manner, while propionate prevents growth in both a pH-dependent and pH-independent manner.

### Ornithine decarboxylase activity partially protects against propionate toxicity

**Fig 2:**
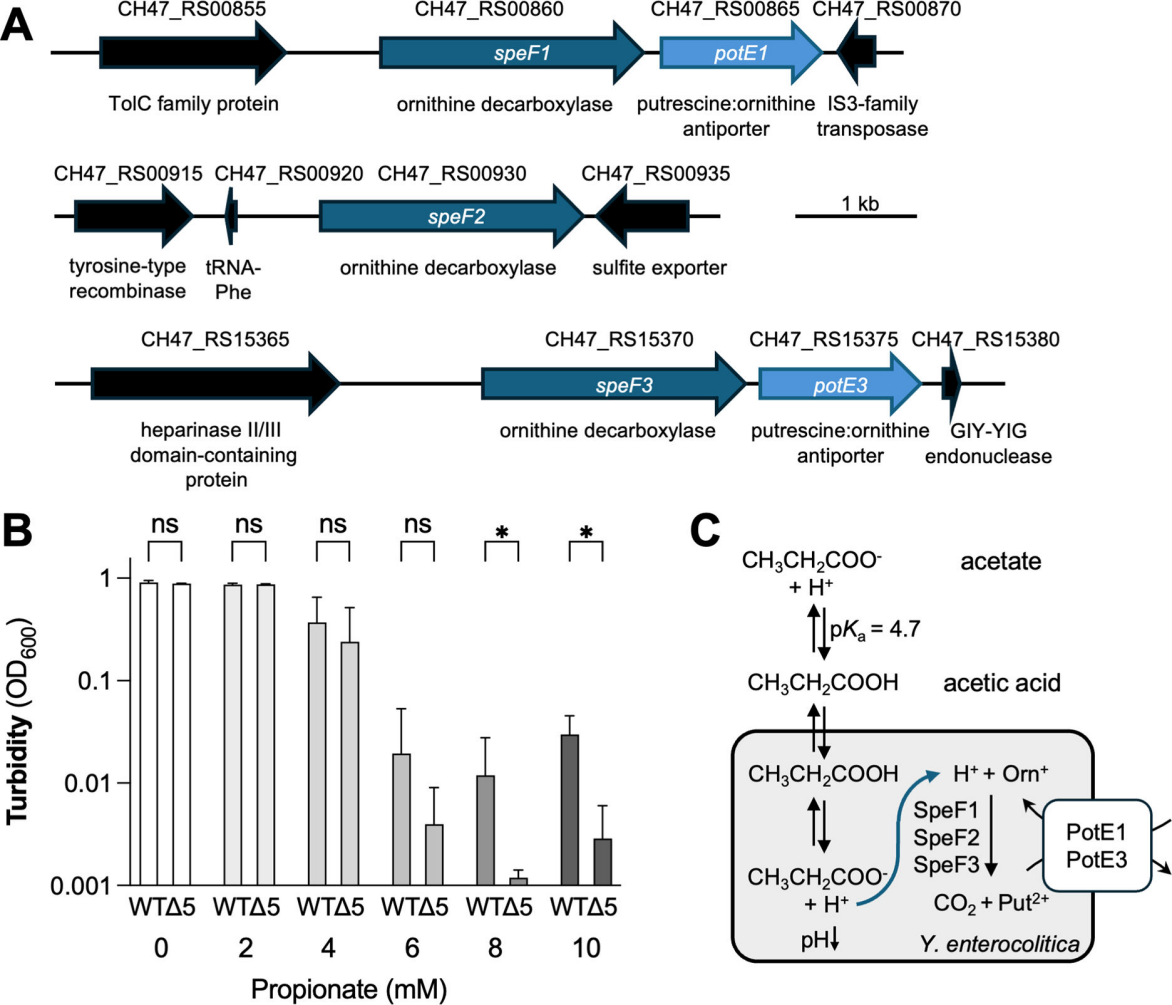
Ornithine decarboxylase activity partially protects against propionate toxicity. (**A**) Schematic representation of the genetic context of the three putative *speF* genes (encoding ornithine decarboxylase) in *Y. enterocolitica*. (**B**) The *Y. enterocolitica* wild-type strain and a mutant lacking all *speF* and *potE* genes (Δ5) were grown in the presence of sodium propionate, as indicated, in LB at a starting pH of 7. Cultures were incubated at 37°C for 24 h, and the absorbance of the culture (OD_600_) was determined. Bars represent the geometric mean, and error bars represent the geometric standard deviation from four independent experiments. ns, not statistically significant; *, *P* < 0.05. Log-normal, two-way ANOVA/Šídák’s multiple comparison test. (**C**) Working model for acidification of the *Y. enterocolitica* cytoplasm by propionate and action of ornithine decarboxylase.

To better understand how propionate restricts *Y. enterocolitica* growth, we investigated known mechanisms of preventing SCFA-mediated cytoplasmic acidification in pathogenic Proteobacteria. *Salmonella* uses the amino acid decarboxylases CadA and SpeF to maintain pH homeostasis when exposed to SCFAs (49–51). We analyzed representative *Y. enterocolitica* genomes and did not identify clear orthologs of the *Salmonella cadA* gene. We therefore focused on the ornithine decarboxylase SpeF. Curiously, the *Y. enterocolitica* genome contains three *speF* genes, as well as two *potE* genes, encoding putative ornithine/putrescine antiporters (Fig. 2A). To determine whether ornithine decarboxylation influences *Y. enterocolitica* growth in the presence of propionate, we generated a mutant lacking all *speF* and *potE* genes (Δ5 = Δ*speF1-potE1* Δ*speF2* Δ*speF3-potE3*) and measured the effect of propionate on the growth of this strain (Fig. 2B). In the absence of propionate, the Δ5 mutant grew to levels similar to the wild-type strain. However, at concentrations of 8 and 10 mM, growth of the Δ5 mutant was significantly reduced compared to the wild-type strain, suggesting that ornithine decarboxylation contributes to growth of *Y. enterocolitica* in the presence of propionate (Fig. 2B), likely through its canonical mechanism of counteracting acidification of the cytoplasm by consuming protons (Fig. 2C).

### A genetic selection assay implicates the Pta-AckA pathway in propionate toxicity

**Fig 3:**
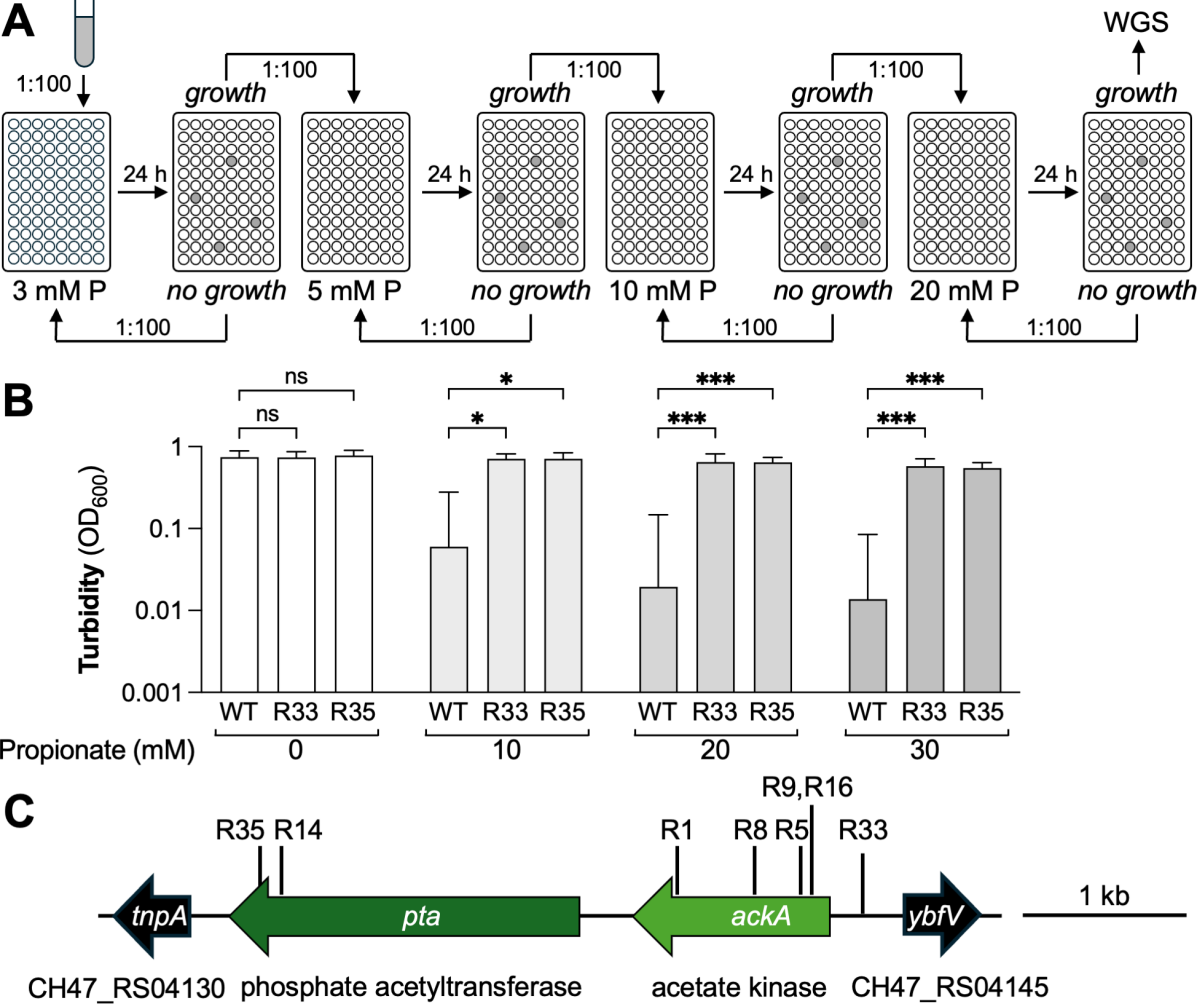
Genetic selection assay to identify genes involved in propionate toxicity. *Y. enterocolitica* WA-314 was cultured in the presence of increasing concentrations of propionate. Single colonies were isolated from cultures that grew in the presence of 20 mM propionate, and their genome sequences were determined. (**A**) Schematic representation of the genetic selection assay; see text for details. (**B**) Representative growth experiment for two independent single colonies (R33 and R35) that were identified as resistant under propionate concentrations that restrict growth of the wild-type (WT) strain. Bacteria were cultured at 37°C in LB supplemented with propionate as indicated, and turbidity was recorded after 24 h. Bars represent the geometric mean, and error bars represent the geometric standard deviation from four independent experiments. ns, not statistically significant; *, *P* < 0.05; ***, *P* < 0.001. Log-normal, two-way ANOVA/Tukey’s multiple comparison test. (**C**) Location of mutations in eight independent strains (R1, R5, R8, R9, R14, R16, R33, and R35) associated with resistance to propionate.

Given that ornithine decarboxylation is insufficient to fully detoxify propionate (Fig. 2B) and that propionate restricts growth in a pH-independent manner (Fig. 1B, E, and H), we focused our attention on other mechanisms and considered the possibility that propionate could be converted to a toxic metabolite. To gain additional insights into propionate toxicity, we performed a selection experiment to identify genes required for propionate growth restriction (Fig. 3). Wild-type *Y. enterocolitica* was cultured in 96-well plates in the presence of sublethal concentrations of propionate (3 mM) in lysogeny broth (LB) to enrich for cells that exhibit a growth advantage under these conditions (Fig. 3A). After several passages, growth was detected in some wells. Partially resistant cultures were exposed to increasing concentrations of propionate until growth at 20 mM propionate was observed. We purified single colonies from eight cultures, and these eight independent isolates were chosen for additional testing. In the absence of propionate, no major growth deficits were noted (Fig. 3B). Growth of these isolates was unimpeded by propionate at concentrations that restrict growth of the wild-type strain (Fig. 3B). We then performed whole genome sequencing on the eight isolates as well as the WA-314 wild type. Curiously, we identified mutations in either *pta* or *ackA* in all our sequenced isolates (Table 1; Table S1; Fig. 3C), suggesting that these genes may be involved in propionate-mediated growth restriction.

**TABLE 1:** Mutations identified in propionate-resistant *Y. enterocolitica* strains

Strain designation	Genes affected	Mutation	Annotation
R1	*ackA sanA*	T→A (TGCACC)_5→6_	L310Q (CTG→CAG) coding (764/768 nt)
R5	*ackA*	G→A	A59T (GCT→ACT)
R8	*ackA*	C→G	T151S (ACT→AGT)
R9	*ackA*	G→A	G32S (GGT→AGT)
R14	*pta tssI*	C→A G→A	A602E (GCA→GAA) P787P (CCG→CCA) (syn)
R16	*ackA*	G→A	A59T (GCT→ACT)
R33	*ackA-ybfV*	G→A	Intergenic region
R35	*pta*	C→G	F648L (TTC→TTG)

### Lack of pta or AckA activity rescues growth of *Y. enterocolitica* in the presence of propionate

**Fig 4:**
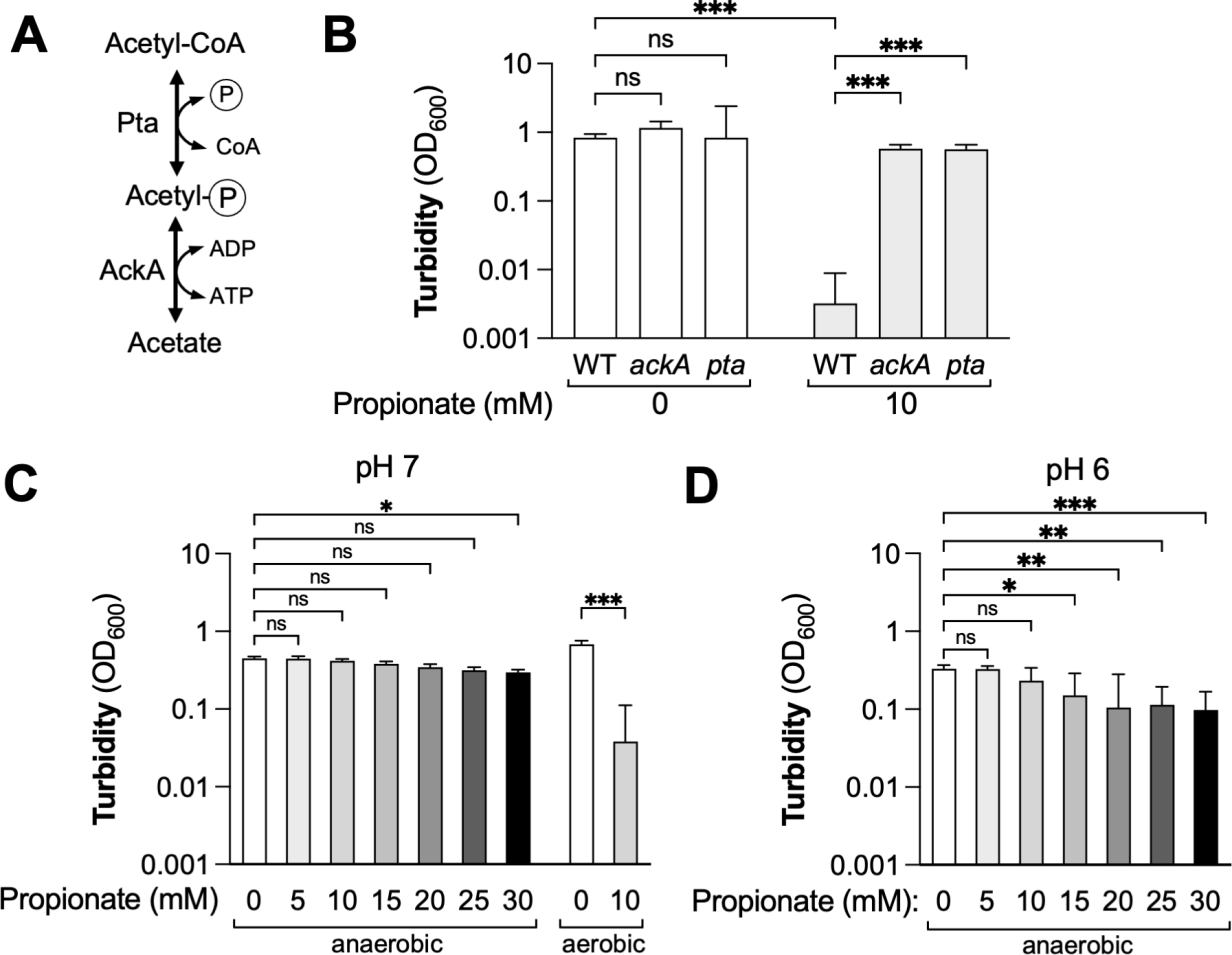
Lack of Pta or AckA activity rescues growth of *Y. enterocolitica* in the presence of propionate. (**A**) The canonical AckA-Pta pathway interconverts acetate and acetyl-CoA. (**B**) Growth of the WA-314 wild-type strain, an isogenic *ackA*, and an isogenic *pta* mutant in media supplemented with propionate. Bacteria were cultured aerobically at 37°C in LB supplemented with propionate as indicated, and turbidity was recorded after 24 h. Bars represent the geometric mean, and error bars represent the geometric standard deviation from four independent experiments. ns, not statistically significant; ***, *P* < 0.001. Log-normal, two-way ANOVA/Tukey’s multiple comparison test. (**C and D**) The *Y. enterocolitica* wild-type strain was cultured anaerobically or aerobically, as indicated, at room temperature in LB at starting pH values of 7 (**C**) and 6 (**D**). Bars represent the geometric mean, and error bars represent the geometric standard deviation from four independent experiments. ns, not statistically significant; *, *P* < 0.05; **, *P* < 0.01; ***, *P* < 0.001. Log-normal, one-way ANOVA/Tukey’s multiple comparison test.

The *pta* and *ackA* genes encode phosphotransacetylase and acetate kinase, the two enzymes that form the Pta-AckA pathway (Fig. 4A). Our suppressor screen mainly revealed point mutations in either the coding sequence or the promoter region of these genes (Table 1; Table S1), which could in principle result in either increased or decreased phosphotransacetylase and acetate kinase activity. The missense mutations we had identified in our screen did not map to known substrate binding sites (52), and current protein structural prediction algorithms such as AlphaFold are not well suited to readily predict the impact of missense mutations on protein stability (53, 54). To determine whether growth in the presence of propionate could be explained by decreased phosphotransacetylase and acetate kinase activity, we generated *pta* and *ackA* mutants using targeted mutagenesis (Fig. 4B). In liquid culture, these mutants grew to levels similar to that of the wild-type strain, suggesting that the Pta-AckA pathway is not essential under these conditions (Fig. 4B). Supplementation with propionate did not inhibit growth of the *pta* and *ackA* mutants, suggesting that a lack of phosphotransacetylase and acetate kinase activity protects against propionate toxicity. It should be noted that none of the mutations we identified were nonsense mutations. Given the importance of the Pta-AckA pathway in bacterial metabolism, it is conceivable that a complete lack of either phosphotransacetylase or acetate kinase activity, as in the case of a nonsense mutation, would slow growth under specific growth conditions (e.g., under the conditions used for the selection). As such, it is possible that any nonsense mutations that may have arisen were selected against and thus not recovered in our experiment.

The Pta-AckA pathway allows for the interconversion of acetyl-CoA and acetate, with acetyl-phosphate as an intermediate (Fig. 4A). The physiological functions of the Pta-AckA pathway are to generate ATP while producing acetate and to consume acetate when acetate is the sole carbon source (55). Purified AckA from *Salmonella* and *Escherichia coli* exhibits promiscuous activity and converts both acetate and propionate to the respective acyl-phosphate (52, 56). Given the substantial amino acid sequence similarity between *Salmonella*, *E. coli*, and *Y. enterocolitica* AckA and Pta enzymes (Fig. S1), we hypothesized that the *Y. enterocolitica* Pta-AckA pathway may catalyze the conversion of propionate to propionyl-CoA. The directionality of the Pta-AckA pathway depends on growth conditions. During fermentation, conversion of abundant acetyl-CoA to acetate allows for substrate-level phosphorylation. We reasoned that, if propionate is converted to propionyl-CoA through promiscuous activity of the Pta-AckA pathway, then propionate would not be toxic under fermentative conditions due to high acetyl-CoA availability, with acetate production being the favored reaction. Consistent with this idea, *Y. enterocolitica* grew readily in the presence of propionate under anaerobic conditions (5% H_2_, 5% CO_2_, 90% N_2_) at pH 7, even at concentrations that were highly restrictive under aerobic conditions (Fig. 4C). Of note, propionate slightly reduced growth of *Y. enterocolitica* in media with a pH of 6 (Fig. 4D), providing additional evidence that propionate exhibits pH-dependent and pH-independent toxicity.

### Exogenous acetate rescues propionate toxicity

**Fig 5:**
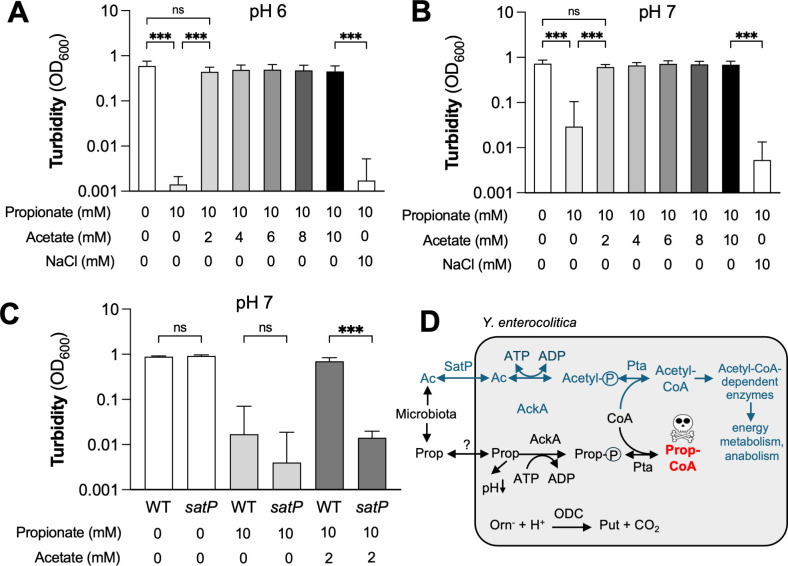
Effect of exogenous acetate on propionate toxicity. (**A and B**) The *Y. enterocolitica* wild-type strain was cultured in LB at pH 6 (**A**) and pH 7 (**B**). Sodium propionate, sodium acetate, and sodium chloride were added as indicated. After 24 h of aerobic incubation at 37°C, the turbidity of the culture was determined. (**C**) The wild-type strain and an isogenic *satP* mutant were cultured as described above in the presence of propionate and acetate, as indicated. (**D**) Working model for propionate toxicity in *Y. enterocolitica* and mechanisms for counteracting toxicity. For details, see text. Ac, acetate; ns, not statistically significant; orn, ornithine; prop, propionate; put, putrescine. Bars represent the geometric mean, and error bars represent the geometric standard deviation from four independent experiments. ***, *P* < 0.001. Log-normal, one-way ANOVA/Tukey’s multiple comparison test.

The *K*_*m*_ values of purified *Salmonella* AckA for acetate and propionate are 1.2 and 11 mM (52), suggesting that there is a preference for acetate over propionate. We therefore hypothesized that addition of acetate to the media might rescue propionate toxicity (Fig. 5A and B). Addition of as little as 2 mM acetate fully restored growth of *Y. enterocolitica* in the presence of 10 mM propionate under aerobic conditions. This rescue was not mediated by changes in osmolarity since supplementation with 10 mM NaCl had no discernible effect on propionate growth restriction (Fig. 5A and B).

We next sought to determine whether acetate needs to be taken up to confer propionate resistance. *Y. enterocolitica* encodes two putative acetate transporters, the succinate-acetate/proton symporter SatP (also known as YaaH) (57, 58) and the acetate permease ActP (also known as YjcG) (59). Since ActP-mediated uptake of acetate is inhibited by propionate (59), we focused our attention on SatP. We created a *Y. enterocolitica* mutant lacking the *satP* gene and analyzed growth in the presence of acetate and propionate (Fig. 5C). In the absence of exogenous monocarboxylates, the *satP* mutant grew to similar levels as the wild-type strain. Addition of 10 mM propionate impeded growth of the wild-type and the *satP* mutant to a similar extent, suggesting that SatP is not required for propionate uptake. As we had seen before (Fig. 5A), exogenous acetate in the media rescued growth of the wild-type strain, while growth of the *satP* mutant was not restored (Fig. 5C). Overall, these results indicate that SatP-mediated acetate uptake rescues propionate toxicity.

## DISCUSSION

The gut microbiome impedes host colonization by enteric pathogens through the production of SCFAs. *Y. enterocolitica* is likely exposed to SCFAs during initial invasion of the small intestine at early stages of infection, when colonizing or passing through the large intestine, and during fecal shedding. In our study, we found that acetate, butyrate, and, most prominently, propionate, impede *Y. enterocolitica* growth *in vitro* in a pH-dependent manner. These findings are consistent with the idea that SCFAs exert some of their effect on *Y. enterocolitica* growth by acidifying the bacterial cytoplasm, a known mechanism of SCFA toxicity (22). In our study, we found that propionate toxicity is in part counteracted by ornithine decarboxylase activity, since a mutant lacking all known ornithine decarboxylase systems was unable to grow in the presence of propionate. This finding is in line with previous reports showing that in murine infection models, ornithine decarboxylase is critical for gut colonization by other enteric pathogens (49, 51). The ability to produce ornithine decarboxylase activity is highly conserved among pathogenic *Y. enterocolitica*, a fact that is useful for biochemical characterization of clinical isolates (60, 61). The *Y. enterocolitica* 8081 and WA-314 genomes encode three *speF* genes (Fig. 3A). Our work leaves unresolved as to whether the gene products fulfill redundant functions.

Propionate exhibited more pronounced inhibitory activity compared to acetate and butyrate. We found that this enhanced toxicity was pH independent and required the Pta-AckA pathway. Based on our genetic data, we propose the following working model (Fig. 5D): propionate enters the cell either by diffusion or through an unknown uptake mechanism. If intracellular propionate concentrations significantly exceed the concentration of acetate, propionate is converted to propionyl-CoA by the Pta-AckA pathway. Formation of propionyl-CoA inhibits bacterial growth, either by consuming ATP, diverting CoA (depleting the CoA pool), or inhibiting CoA-dependent enzymes. It is also plausible that a combination of these effects is needed to arrest growth. Key aspects of our model are supported by findings in other microorganisms. First, CoA-dependent enzymes, such as AckA, Pta, and acetyl-CoA synthase, are well documented to exhibit promiscuous substrate specificity and readily interconvert propionate to propionyl phosphate or propionyl-CoA (52, 56, 62–64). This promiscuity of AckA and Pta enables *Salmonella* to degrade the toxic metabolite alpha-ketobutyrate via propionyl-CoA and propionyl-phosphate to propionate (65). Second, propionyl-CoA is known to exert its toxicity by inhibiting central metabolism enzymes. In *Rhodobacter sphaeroides* and *Streptococcus faecalis*, propionyl-CoA inhibits pyruvate dehydrogenase (66, 67). Similarly, propionyl-CoA inhibits the *E. coli* citrate synthase (68). Propionate also prevents growth of *Aspergillus nidulans*; in this organism, propionyl-CoA inhibits the activity of ATP-dependent citrate lyase, succinyl-CoA synthetase, and pyruvate-dehydrogenase (69). Third, butyrate inhibits *Clostridium difficile* growth through a “backflow mechanism” that involves CoA-dependent enzymes (70, 71). When butyrate is high in the environment, butyrate passes into the *C. difficile* cell and enters a reversed butyrogenesis pathway. As such, exogenous butyrate is converted to crotonyl-CoA and hydroxybutyryl-CoA, rewiring a metabolic pathway from its canonical function of energy production to energy consumption (71). A key difference between the mechanism of butyrate toxicity in *C. difficile* and propionate toxicity in *Y. enterocolitica* is the fact that the former is the reversal of an existing biochemical pathway, while the latter involves promiscuous activity of the PtA and AckA enzymes. Purified *E. coli* AckA is “specific” for propionate and acetate, but not lactate, succinate, or butyrate (56), which may explain why we did not observe pH-independent butyrate toxicity in *Y. enterocolitica*.

Bona fide pathogens have evolved mechanisms to overcome SCFA-mediated colonization resistance, and these mechanisms are well documented for *C. difficile* and Enterobacteriaceae family members (16, 72, 73). *Salmonella* can circumvent propionate intoxication in the MCC by coupling propionate degradation to anaerobic respiration (38). *Mycobacterium tuberculosis* employs the MCC pathway found in *S*. Tm (74), the methylmalonyl pathway (75) which converts propionyl-CoA to succinyl-CoA, and directly uses propionyl-CoA for the biosynthesis of membrane lipids (76, 77). Based on publicly available genome sequences, *Y. enterocolitica* is predicted to lack key enzymes of the MCC and methylmalonyl pathways, raising the question as to how *Y. enterocolitica* overcomes propionate-mediated toxicity. We found that propionate toxicity can be suppressed by exogenous acetate (Fig. 5D). As such, high levels of acetate, either produced intracellularly through fermentation or present in the environment, negate the effects of propionate toxicity. One limitation of our work is that we explored propionate toxicity under laboratory conditions and not in the mammalian gut. Given that typical acetate concentrations in the mammalian gut are substantially higher than propionate levels (41, 42), it is plausible that propionate toxicity is abrogated when *Y. enterocolitica* is exposed to SCFA in the mammalian gut. This finding may explain why *Y. enterocolitica* does not require canonical propionate detoxification systems, such as the MCC, to deal with pH-independent propionate toxicity. While likely not relevant for *Y. enterocolitica* infection, propionate toxicity, possibly mediated through the Pta-AckA pathway, may play a role for host-microbe interactions on the skin. In sebum, produced by sebaceous glands in hair follicles, propionate is the most abundant SCFA (78, 79), and thus propionate could affect the growth of Pta and AckA-expressing members of the skin microbiome. Of note, calcium propionate (E282) is a common food preservative that prevents microbial growth, primarily in baked goods (80–82). Since the propionate toxicity we observed is based on a fundamental biochemical property of the PtA and AckA enzymes, common among microbes, it may be of interest to determine whether inadvertent propionate conversion to propionyl-CoA through the Pta-AckA pathway is the basis for the efficacy of calcium propionate as a food additive.

## MATERIALS AND METHODS

### Bacterial strains

The bacterial strains and mutants used in this work are shown in Table 2. All *Y. enterocolitica* mutants were generated in the WA-314 (ATCC 51871) wild-type strain (serotype O:8). For routine culture, we grew *E. coli* and *Y. enterocolitica* in LB (10 g/L tryptone, 5 g/L yeast extract, and 10 g/L sodium chloride) or on LB plates (10 g/L tryptone, 5 g/L yeast extract, 10 g/L sodium chloride, and 15 g/L agar) at 37°C or 27°C, respectively. Kanamycin (100 mg/L) and nalidixic acid (50 mg/L) were added to LB and LB plates, as indicated.

**TABLE 2:** Strains used in this study

Strain	Genotype	Source or reference
*E. coli*		
DH5α λ*pir*	F^-^ *endA1 hsdR17* (r^-^m^+^) *supE44 thi-1 recA1 gyrA relA1* D(*lacZYA-argF*)*_U189_* f80*lacZ*ΔM15 λ*pir*	(83)
S17-1 λ*pir*	C600::RP4 2-(Tet::Mu) (Kan::Tn7) λ*pir recA1 thi pro hsdR* (r^-^ m^+^)	(84)
*Y. enterocolitica*		
WA-314	Wild-type strain (serotype O:8)	ATCC 51871
MM114	WA-314 *ackA*::pMM108	This study
MM115	WA-314 *pta*::pMM106	This study
MM92	WA-314 Δ*speF1-potE1*	This study
MM100	WA-314 Δ*speF1-potE1* Δ*speF2*	This study
MM103	WA-314 Δ*speF1-potE1* Δ*speF2* Δ*speF3-potE3*	This study
MM126	WA-314 Δ*satP*	This study

### Targeted mutagenesis in *Y. enterocolitica*

Gibson assembly cloning was used to generate the suicide plasmids listed in Table 3. For generating clean deletions, flanking regions surrounding the coding sequence of the gene of interest were identified, while for insertional mutants, a DNA region within the first third of the coding sequence was identified. Primer sequences were generated using the NEBuilder Assembly Tool v.2.10.3 and are listed in Table 4. DNA regions of interest were amplified by PCR using WA-314 as the template and then assembled using a commercial Gibson assembly mix (New England Biolabs). Assembled plasmids were transformed into *E. coli* DH5α λ*pir*. Positive clones were identified by PCR and DNA sequencing of the insert. The plasmid of interest was purified (Qiagen Midi DNA Plasmid Extraction kit) and then transformed into *E. coli* S17-1 λ*pir*. S17-1 λ*pir* harboring plasmids of interest was used as the donor strain for conjugation into *Y. enterocolitica* WA-314. The conjugation mixture was plated on LB agar plates and incubated at room temperature. After conjugation, single crossover mutants were identified by spread plating on LB plates supplemented with nalidixic acid and kanamycin. To generate MM114 (*ackA*::pMM108) and MM115 (*pta*::pMM106), the plasmids pMM108 and pMM106 were integrated into the WA-314 genome. To generate clean unmarked deletion mutants, we used a sucrose counter-selection strategy. Mutants in which a single crossover event had occurred were cultured in LB at room temperature, and a sample was plated on sucrose plates (5% sucrose, 15 g/L agar, and 8 g/L nutrient broth base). Sucrose-resistant colonies indicated that a second crossover event had taken place. To generate MM126 (Δ*satP*), plasmid pMM146 was used to create a clean deletion of the *satP* genes in WA-314. MM103 (Δ*speF1-potE1* Δ*speF2* Δ*speF3-potE3*) was generated by sequentially integrating and removing plasmids pMM90, pMM96, and pMM97 from the chromosome, creating MM92 (Δ*speF1-potE1*) and MM100 (Δ*speF1-potE1* Δ*speF2*) as intermediates. Insertional and clean deletion mutants were screened and confirmed by PCR.

**TABLE 3:** Plasmids used in this study

Plasmids	Relevant characteristics	Source or reference
pGP705	*ori*R6K *mobRP4* Kan^R^	(85)
pGP706	*ori*R6K *mobRP4 sacRB* Kan^R^	(85)
pMM108	Internal fragment of *ackA* in pGP705	This study
pMM106	Internal fragment of *pta* in pGP705	This study
pMM90	Upstream region of *speF1* and downstream region of *potE1* in pGP706	This study
pMM96	Upstream and downstream region of *speF2* in pGP706	This study
pMM97	Upstream region of *speF3* and downstream region of *potE3* in pGP706	This study
pMM146	Upstream and downstream region of *satP* in pGP706	This study

**TABLE 4:** Oligonucleotides used in this study

Target	Nucleotide sequence
Δ*speF1-potE1* deletion mutant	5′-TCTTCTAGAGGTACCGCATGGGAAATCACACCGCAACG-3′ 5′-TCTGCCAGCGGCTAAGCATGATCACAGAATTAATTTC-3′ 5′-CATGCTTAGCCGCTGGCAGATAACCTGC-3′ 5′-GGAGAGCTCGATATCGCATGTTGCAGGACAGGACACTC-3′
Δ*speF2* deletion mutant	5′-TCTTCTAGAGGTACCGCATGTGCTAACTATTTTTACAATCCATG-3′ 5′-TGTTTTCAGAGATATCTTAGACATACACGTC-3′ 5′-CTAAGATATCTCTGAAAACAGATAGACAG-3′ 5′-GGAGAGCTCGATATCGCATGCACTATTTATCCCCGTAG-3′
Δ*speF3-potE3* deletion mutant	5′-TCTTCTAGAGGTACCGCATGCATGACATTCGTCACAGAATG-3′ 5′-ATTCGATTATTATGAACCCGTCCAACATTTTTG-3′ 5′-CGGGTTCATATAATCGAATGGTTCATATTGAGC-3′ 5′-GGAGAGCTCGATATCGCATGAGTGGTGGTATTAGGCGATC-3′
Δ*satP* deletion mutant	5′-TCTTCTAGAGGTACCGCATGCAGCTGTTGGTTCGTTGATG-3′ 5′-TGAACTGTAAGGCCCCATCTTATTCCCAAC-3′ 5′-AGATGGGGCCTTACAGTTCACTGCGAAAAATTAC-3′ 5′-GGAGAGCTCGATATCGCATGTCTTGCGTCGTTCCAGTATTTG-3′
*ackA* insertional mutant	5′-TCTTCTAGAGGTACCGCATGATTTGCTATCATCGACGC-3′ 5′-GGAGAGCTCGATATCGCATGGTCAAAGACTGCAACATTTTTATC-3′
*pta* insertional mutant	5′-TCTTCTAGAGGTACCGCATGGGATCGATGCTCGCGACAC-3′ 5′-GGAGAGCTCGATATCGCATGTGTCATCCGTTCCATGGAAC-3′

### SCFA growth inhibition assays

*Y. enterocolitica* was grown in LB statically overnight at room temperature. The turbidity at a wavelength of 600 nm (OD_600_) was determined with an Eppendorf BioSpectrometer and used to make stock cultures of 10^8^ CFU/mL in fresh LB. LB was then adjusted with HCl or NaOH to reach the desired pH indicated in the assays. Media containing the indicated concentrations of sodium acetate, sodium butyrate, and/or sodium propionate were then inoculated with 10^6^ CFU/mL of bacteria from stock cultures. The cultures in round-bottom glass test tubes were incubated at the indicated temperatures for 24 h, and the OD_600_ of each culture was determined. Anaerobic growth assays were carried out in an anaerobic chamber (90% N_2_, 5% CO_2_, 5% H_2_; Sheldon Manufacturing). For data analysis, the limit of detection for the OD_600_ assays was set to 0.001.

### *In vitro* screen for propionate resistance

*Y. enterocolitica* was grown in LB overnight standing at room temperature. The turbidity (OD_600_) was determined and used to make a stock culture of 10^8^ CFU/mL in LB. This was used to inoculate LB medium containing 3 mM sodium propionate. The media at the beginning of the experiment were neutral with a pH of 7. The culture was then plated into 96-well round-bottom plates and incubated at 37°C for 24 h. A subset (0.002 mL) of each well’s culture was then transferred into a fresh 96-well plate well containing 0.2 mL of LB medium with 3 mM sodium propionate and incubated at 37°C for 24 h. Subsequent passages were then carried out until notable growth was observed in some of the wells. Then the sodium propionate concentration was increased in a stepwise fashion for each subsequent passage (5, 10, and 20 mM). Propionate-resistant strains of *Y. enterocolitica* characterized by growth in these wells were then plated on LB medium, and single colonies were picked for purification.

### Whole genome sequencing of propionate-resistant strains

Propionate-resistant strains and the WA-314 wild-type strain were whole genome sequenced using SEQ Center’s Illumina Whole Genome Sequencing Service. Samples were submitted in accordance with SEQ Centers Sample Submission Guidelines. *Y. enterocolitica* genomic DNA was generated using the Qiagen DNeasy PowerLyzer Microbial Kit per the manufacturer’s instructions. Bioinformatic analysis and SNP variant calls against the published 8081 genome were provided by SEQ Center. Illumina-generated 2 × 151 bp paired-end read data were used as the input for variant calling. Variant calling was carried out using Breseq v.0.37.1 under default settings (86). Variant SNPs were then manually compared against SNPs found in the WA-314 strain to determine true SNP variants associated with propionate resistance (Table 1).

### Data analysis and statistics

DNA and primary amino acid sequences were analyzed and plotted using MacVector (MacVector, Inc) v.17.5.6. Multiple sequence alignment of primary amino acid sequences was performed using the ClustalW algorithm in MacVector. Experimental data were analyzed and processed in Microsoft Excel and GraphPad Prism v.10.6.1. All raw data were transformed with the natural logarithm prior to statistical analysis to ensure that the data were normally distributed. To determine statistical differences between groups, a one-way or two-way ANOVA test and post hoc test was applied to the logarithmically transformed data. The type of ANOVA and post hoc test is specified in the figure legends. *P* values less than 0.05 were considered significant. ns denotes not statistically significant (*, *P* < 0.05; **, *P* < 0.01; ***, *P* < 0.001).

## Data Availability

Raw sequence reads and associated metadata are available under accession number PRJNA1378261 in the NCBI sequence read archive.

## SUPPLEMENTAL MATERIAL

Fig. S1 iai.00043-26-s0001.pdf Sequence alignment (A) for the primary amino acid sequences of AckA and Pta from *Y. enterocolitica*, *S. Typhimurium*, and *E. coli*, and (B) identity and similarity scores.

Table S1 iai.00043-26-s0002.xlsx Genetic variants identified by whole genome sequencing after selection in the presence of increasing concentrations of propionate.

ASM does not own the copyrights to Supplemental Material that may be linked to, or accessed through, an article. The authors have granted ASM a non-exclusive, world-wide license to publish the Supplemental Material files. Please contact the corresponding author directly for reuse.
