# Supplementary material for: Acetate uptake alleviates propionate-mediated growth restriction in Yersinia enterocolitica
Source: Infect Immun. 2026 Apr 15;94(5):e00043-26. doi: 10.1128/iai.00043-26 (PMC13163199; doi:10.1128/iai.00043-26)
Supplement: Fig. S1 — Sequence alignment (A) for the primary amino acid sequences of AckA and Pta from Y. enterocolitica, S. Typhimurium, and E. coli, and (B) identity and similarity scores. [file iai.00043-26-s0001.pdf]

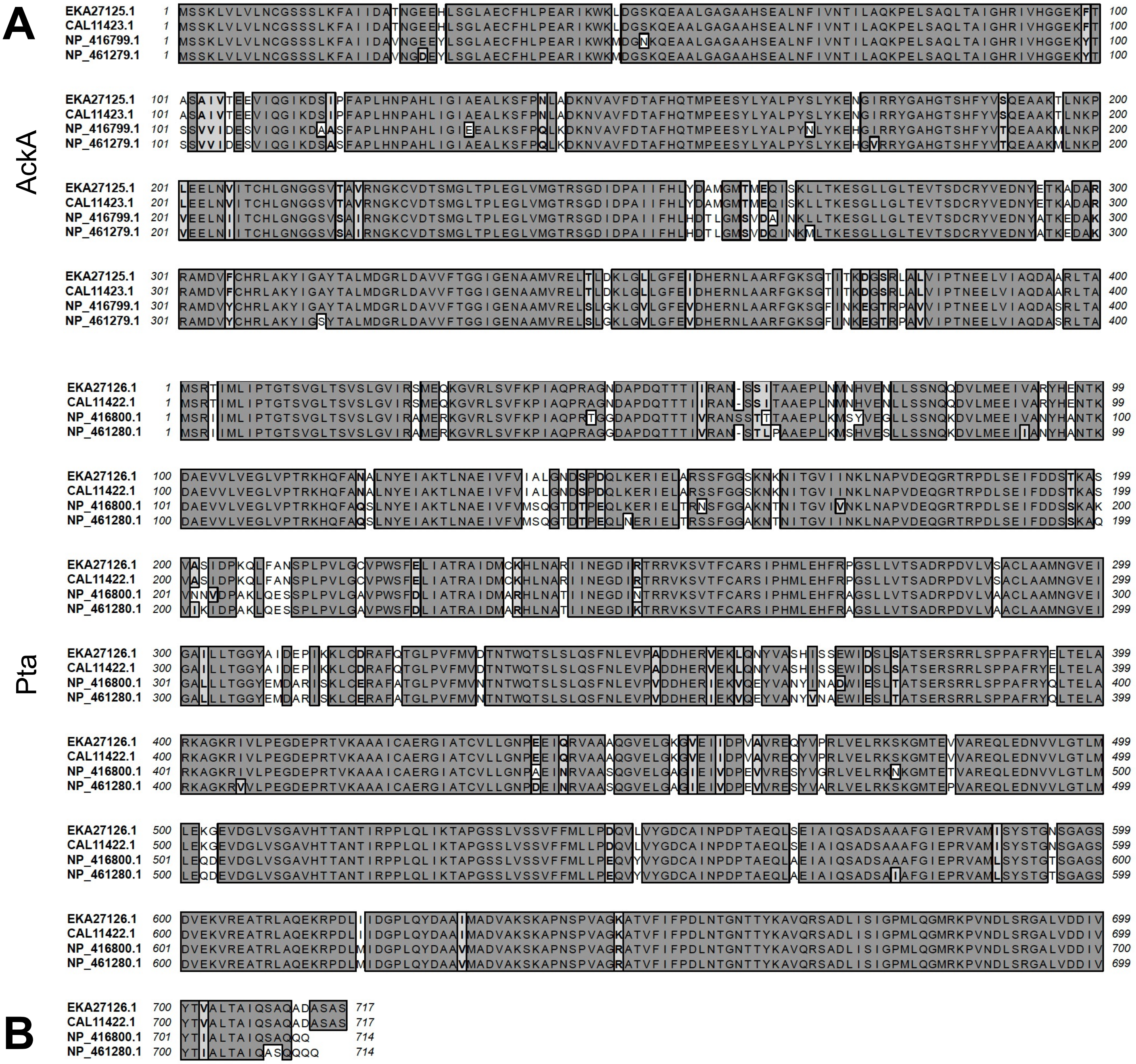

B

**AckA**		EKA27125.1	CAL11423.1	NP_461279.1	NP_416799.1
Y. ent WA-314	EKA27125.1	-	100	88.2	88.0
Y. ent 8081	CAL11423.1	*100*	-	88.2	88.0
S. Tm LT2	NP_461279.1	*96.0*	*96.0*	-	97.8
E. coli K-12	NP_416799.1	*95.0*	*95.0*	*99.0*	-

Identity Score

Similarity Score

**Pta**		EKA27126.1	CAL11423.1	NP_416800.1	NP_461280.1
Y. ent WA-314	EKA27126.1	-	100	86.5	86.5
Y. ent 8081	CAL11423.1	100	-	86.5	86.5
S. Tm LT2	NP_416800.1	*93.3*	*93.3*	-	96.4
E. coli K-12	NP_461280.1	*93.7*	*93.7*	*97.8*	-

Identity Score

Similarity Score

Supplementary Figure S1: Sequence alignment (A) for the primary amino acid sequences of AckA and Pta from *Y. enterocolitica* (*Y. ent*), *Salmonella* Typhimurium (*S.* Tm), and *E. coli.* (B) Identity and similarity scores. Multiple alignment was performed using the ClustalW algorithm.
